# Perspectives on the Use of Liquid Extraction for Radioisotope Purification

**DOI:** 10.3390/molecules24020334

**Published:** 2019-01-18

**Authors:** Petra Martini, Andrea Adamo, Neilesh Syna, Alessandra Boschi, Licia Uccelli, Nopphon Weeranoppanant, Jack Markham, Giancarlo Pascali

**Affiliations:** 1 Department of Morphology, Surgery and Experimental Medicine, University of Ferrara, Via Luigi Borsari, 46-44121 Ferrara, Italy; petra.martini@unife.it (P.M.); alessandra.boschi@unife.it (A.B.); licia.uccelli@unife.it (L.U.); 2 Legnaro National Laboratories, Italian National Institute for Nuclear Physics (LNL-INFN), Viale dell’Università, 2, 35020 Legnaro (PD), Italy; 3 Zaiput Flow Technologies, 300 2nd Avenue, Waltham, MA 02451, USA; aadamo@zaiput.com; 4 ANSTO Minerals, New Illawarra Rd, Lucas Heights, NSW 2234, Australia; neileshs@ansto.gov.au; 5 Department of Chemical Engineering, Faculty of Engineering, Burapha University, 169 Longhard Bangsaen, Saensook, Muang, Chonburi 20131, Thailand; nppn2188@gmail.com; 6 ANSTO National Research Cyclotron, 81 Missenden Rd, Camperdown, NSW 2050, Australia; jackm@ansto.gov.au; 7 Brain and Mind Centre, University of Sydney, 94 Mallett St, Camperdown, NSW 2050, Australia

**Keywords:** liquid-liquid extraction, radioisotopes, radiometals, nuclear medicine, purification, separation

## Abstract

The reliable and efficient production of radioisotopes for diagnosis and therapy is becoming an increasingly important capability, due to their demonstrated utility in Nuclear Medicine applications. Starting from the first processes involving the separation of ^99m^Tc from irradiated materials, several methods and concepts have been developed to selectively extract the radioisotopes of interest. Even though the initial methods were based on liquid-liquid extraction (LLE) approaches, the perceived difficulty in automating such processes has slowly moved the focus towards resin separation methods, whose basic chemical principles are often similar to the LLE ones in terms of chelators and phases. However, the emerging field of flow chemistry allows LLE to be easily automated and operated in a continuous manner, resulting in an even improved efficiency and reliability. In this contribution, we will outline the fundamentals of LLE processes and their translation into flow-based apparatuses; in addition, we will provide examples of radioisotope separations that have been achieved using LLE methods. This article is intended to offer insights about the future potential of LLE to purify medically relevant radioisotopes.

## 1. Introduction

Personalization of diagnostic and curative options is becoming an important target in modern medicine. To achieve such results, there is an increasing need of a large availability of flexible and diversified tools. Nuclear Medicine is also experiencing this drive, more evident in the design of theranostic tracers [1]. In the most common acceptation, such approach requires the use of a biomolecular targeting entity conjugated with an appropriate chelating moiety; this functionality is then used to chelate radiometals with either diagnostic or curative properties, thus realizing a theranostic tracer. This innovative concept has been one of main contributors to a renewed focus on the efficient and wide availability of radiometals with a large spectrum of emissive properties. Such radioisotopes can be produced with a variety of approaches, all having in common the selective extraction of the radiometal of interest from a radioactively irradiated material (e.g., by particle accelerator or reactor). In this perspective paper we will report the features of extractive processes based on liquid-liquid extraction (LLE), whose chemical fundamentals also often represent the basis of resin-based separation approaches. Even though LLE systems have been employed in historical applications, thus accumulating a large body of evidence on the critical parameters and working conditions, LLE has been mostly replaced with resin-based approaches, due to its perceived difficulty of automation and poor reliability with continued use. In this work we also describe how the use of new flow-chemistry methodologies already contributes to increasing the reliability and efficiency of LLE processes in the chemical industry, thus providing the basis for a renewed interest in LLE for radioisotope production.

## 2. Features of Liquid-Liquid Extraction (LLE)

### 2.1. Fundamentals of Batch Extraction

Industrial solvent extraction (also known as liquid-liquid extraction, LLE) was pioneered for the separation and recovery of radioactive materials in the 1940’s [2,3,4]. LLE is a widely used separation process within the chemical, food, hydrometallurgical, nuclear, petrochemical and pharmaceutical industries, mainly due to its cost effectiveness [5]. These industries typically utilise mixer-settlers, centrifuges and (static or agitated) columns. The two former unit types generally represent batch contact with one theoretical transfer unit (NTU) per stage whereas the latter unit type exhibits greater than one NTU and is more similar to flow extraction cases.

LLE involves the mixing of two immiscible liquids and maintaining the droplets (or films) of the dispersed phases to enable materials transfer followed by the separation of the two phases from each other [5]. LLE process is dependent on the composition and the chemistry of the aqueous phase as well as the reaction mechanisms that drive the exchange of target metal onto the organic phase [4]. Exchange mechanisms include chelation/compound formation, ion association and solvation of target metal. Change in aqueous solution pH greatly affects the metal hydrolysis constants that vary with ionic strength, which can also lead to steric and/or kinetic effects. Adjustment of aqueous solution pH can be used to regulate the extraction process [4]. Aqueous phase composition plays an important role when the extraction mechanism is based on ion association and dissociation, as well as solvation. These systems use strong acid or high salt concentrations [4].

Batch extractions of target metal by the solvent is typically characterised by the parameters of distribution coefficient, selectivity, capacity and kinetics. Distribution coefficient (*K_d_*) is the stoichiometric concentration ratio of the target metal between the organic and aqueous phase at equilibrium, as represented in the equation below which is generally used to measure the extent of extraction [6]:

(1) Kd=CorgCaq

*K_d_* values are not constant and will become smaller as the organic phase approaches saturation [7]. Typically, a high *K_d_* (> 10) is desired for the target metal with low *K_d_* values for the impurities. This is also a function of the adopted experimental conditions; e.g. temperature, metal concentrations and phase volume ratios [4]. Low *K_d_* (~1) can be utilized, but at higher solvent phase ratio [3].

The relative quantity of the target metal transferred from the aqueous to the organic phase is referred to as the percent extraction (*%E*), and this is calculated for given *K_d_* and *P*, as described in the equation below, where *P* denotes the volumetric ratio of the two (organic over aqueous) phases. Stage efficiency represents the amount of target metal extracted in a given stage to that which would be extracted under ideal conditions [7]:

(2) $$\%E=\frac{100\;\left(P\right)K_{d}}{1+\left(P\right)K_{d}}$$

Based on this equation, for a system with a given *K_d_*, the percent extracted can be improved by increasing *P* [6]. Other factors that can influence *%E* include extractant concentration, temperature, pH, metal complexation in both the aqueous and organic phases and the metal concentration in the aqueous phase [4].

The separation factor (β) numerically represents the selectivity of the organic phase for the target metal in the aqueous solution compared to the impurities, which is a ratio of the *K_d_* values of the two metals of interest (*a* and *b*), as shown below:

(3) β=Kd(a)Kd(b)

A high β does not necessarily indicate a practical system unless the individual *K_d_* are reasonably large [7]. This parameter can be enhanced through judicious selection of operating conditions, such as extractant concentration, temperature and contact time [2].

The total amount of metal which may be held by a given organic phase is termed saturation capacity and this value establishes the flow of the two phases. If the solvent loading capacity is low and the *K_d_* is high, a higher organic flow ratio could be adopted [3]. Augmenting the extractant concentration can increase the saturation capacity of the organic phase but care must be taken to ensure the extractant remains soluble, otherwise addition of a phase modifier becomes necessary in the organic phase makeup. These additions also increase the operating costs of the separation process. 

The kinetics of the system governs the throughput of the separation process. Fast kinetics are desirable to enable rapid transfer of the target metal and limit the co-extraction of impurities. The kinetics are dependent on the reaction type (e.g., ionic-type reactions are rapid whereas chelate formation reactions can be longer), viscosity of the phases, mixing time and the temperature of the system [4]. The rate of extraction will be dependent on the surface area of the dispersed phase (or droplet size), which is contingent on the amount of energy deployed. However, too much energy can result in stable emulsions (droplets size of < 1 µm) which can slow the extraction rate by limiting mass transfer between the aqueous-organic interface [3,4].

### 2.2. Flow Extraction

Flow extraction commonly refers to a continuous liquid-liquid operation in which different solute components are transferred between two or more phases. Typically, efficient contact between an originating solution and an extracting solvent has to be achieved, and mass transfer takes place while the streams are flowing in a pre-designed channel network. At the end of the flow contacting process (i.e., output stream), if the mass transfer has been efficient, the two phases will altered solute concentrations; in particular, the extracting solvent will be enriched of the extracted component and the originating phase will be consequently depleted of it.

Extraction in microscale flows has some advantages with respect to other approaches. At microscale, the mass transfer is rendered more efficient by the fact that the characteristic diffusion length is much smaller than that in conventional batch operation [8]. As a result, a complete extraction can be achieved more rapidly than a batch extractor, and an approximately 1000-fold increase in mass-transfer coefficient (k_L_a) value has been reported in some applications [5,9], when compared to the equivalent batch processes. Furthermore, the interfacial surface-to-volume (S/V) ratio, another important design parameter for efficient extraction, is greatly improved in microfluidic devices. For instance, traditional agitation, packed bed, or spraying provide a S/V ratio up to 32–450 m^2^/m^3^, while a standard droplet flow regime can reach S/V ratio as high as 5000 m^2^/m^3^ [10,11]. Another advantage of extraction in flow is the mitigation of the reliability issues coming from the wide distribution of droplet sizes typically observed in stirred tank systems. The liquid-liquid interface in the flow extraction is often well-defined and controllable, resulting in fast optimization and robust and reproducible processes. Overviews about microflows and microfluidic extraction have recently been the subject of review articles [12,13].

**Figure 1:**
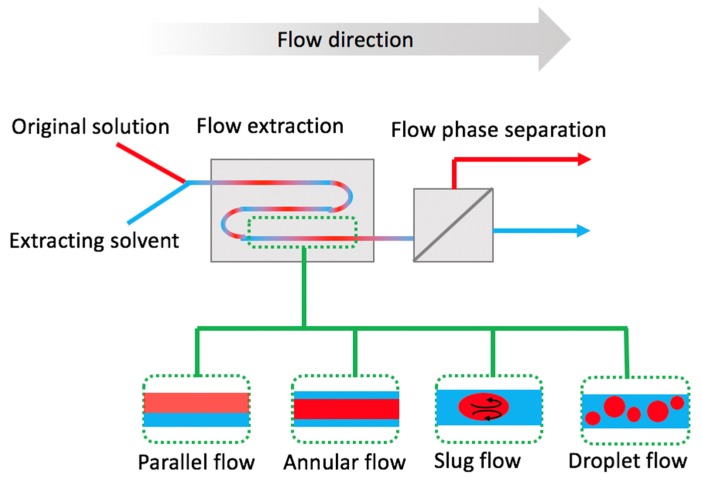
Flow setup consisting of flow extraction and phase separation. In flow, immiscible phases exhibit different flow regimes, including parallel, annular, slug (with internal circulation indicated) and droplet regime.

Liquid-liquid microflows can lead to different types of flow regimes depending on specific flow conditions as shown in Figure 1. Parallel, annular, slug, and droplet flows [14,15] can be observed as flow rates, liquid properties and phase ratios are altered. Inertial, viscous, and interfacial forces are the three main parameters that define the flow regime in different setups. For instance, if viscous and inertial forces dominate over the interfacial force, then parallel flow is observed [16]. Flow maps have been reported by several authors [17,18] to predict the extraction regime solely based on flow parameters and solvent features. Modifications to the fluidic network, such as wall coatings that improve wetting, provide a further control on the extraction regime [19]. Alternatively, pillar or porous structures can be implemented to stabilize the interface [20,21,22]; as an example of this approach, Chang et al. have reported use of helix wires to stabilize a coaxial flow [23]. In this application, the operating window (1–3000 µL/min was reported) depends on the pitch of the wires, whereas higher flow rates can be achieved using smaller pitch.

Among the different regimes, slug flow offers a larger S/V ratio than the parallel flow, and it is by far the most used approach. Friction at the wall causes circulation inside each slug (i.e., Taylor vortex), that increases mass transfer by continuously refreshing the contact interface, and therefore improves the overall efficiency of the extraction [24]. Addition of inert gas as a third flow phase has been shown to enhance the extraction in the slug flow regime as the gas modifies the flow pattern, leading to an increased interfacial area between the liquid phases [25]. Channel size could also affect the extraction performance in the slug flow [26], with a smaller diameter typically linked to improved efficiency. Slug flow naturally has a stable interface, and it can be used effectively to operate in a wide range of flow rates. Currently, slug flow is frequently used in mL/min scale in simple flow contactor devices, such as polymer tubing [27] and coiled flow inverters [28].

The advantages of flow extraction have led to considerable attentions over the last two decades. As a result, flow extraction has been employed for numerous applications including extraction of metals [29,30,31], extraction of rare earth elements [32], microextraction of radionuclides [33,34], purification of nanoparticles [35], separation of catalysts and phase-transfer agents [36], and flow chemistry [37,38,39,40]. Recently, Pedersen et al. applied flow extraction for the extraction of the titanium-45 radioisotope [41].

## 3. Feature of Phase Separation

### 3.1. Batch Phase Separation

Batch phase separation is accomplished when the two immiscible phases disengage by sedimentation and coalescence (growth of small droplets into larger drops); such separation is obtained exploiting gravity or applying external forces, using settlers and centrifuges, respectively [2,3]. Ideally, rapid and complete separation of the organic from the aqueous phase is desired as this governs the equipment size and throughput for industrial applications [4,7]. Factors that influence the rate and completeness of phase disengagement are droplet size, difference in the density of the two phases, viscosity of the two phases, pH of the aqueous phase, temperature of the system, interfacial tension and the presence (or absence) of solids [2,3,4].

Usually phase separations are more successful when the organic phase is continuous (i.e., aqueous phase is dispersed) and recycles can be applied into the mixer to maintain phase continuity [7]. The organic phase must also have good stability (not decompose under conditions of stress, e.g., radioactivity) and also have low aqueous solubility to limit organic losses [4].

### 3.2. Flow Phase Separation

**Figure 2:**
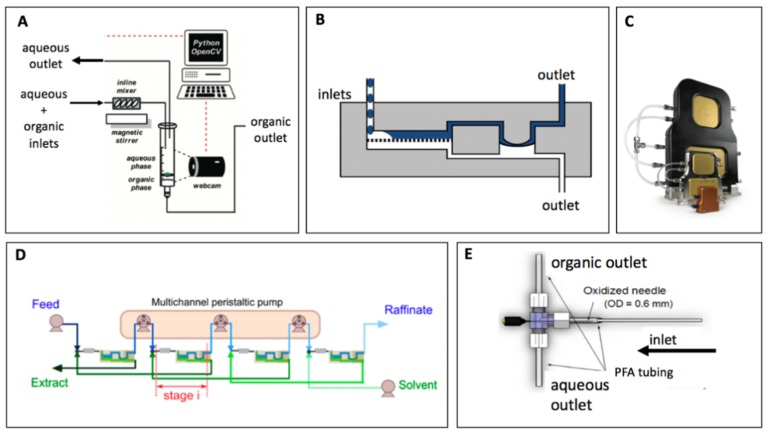
(**A**) a computer-vision gravity-based phase separation, (**B**) Membrane-based separator with self-tuning element for monitoring pressure difference, (**C**) Zaiput’s liquid-liquid separator for continuous separation, (**D**) Arrangement of membrane-based separators for multistage extraction, (**E**) a simple phase separator using hydrophobic junction and hydrophilic needle (Adapted from [27,53,57]).

Flow phase separation can be achieved also using two main forces: gravity or surface friction. Gravity-based separation is similar to the operation in batch systems in which phases settle into different layers, depending on their densities. A setup for separation in flow essentially replicates a larger scale batch operation with the addition of an incoming feed stream and two outgoing separated streams. Successful operation requires control over the flow rate of the outgoing streams. This flow control can be achieved by monitoring the position of the interface between the phases inside the settling tank. For example, Kumar et al used jack-leg type interface controller to monitor the flow rate at the bottom outlet [42]. O’Brien et al. developed a computer-vision approach with webcam and Python coding (Figure 2A) to dynamically adjust pump flowrates to maintain the interface level [43] at a stable position. This simple setup may be a practical tool for laboratory-scale flow synthesis [44]. 

As the dimension of the separation setup decreases, surface forces may start dominating over gravitational forces as indicated by the dimensionless Bond number [17]. Consequently, it is difficult to miniaturize a gravity-based setup, and hence other examples of small-scale flow separations rely on surface forces. To exploit this phenomenon, porous capillaries or membranes are utilized [27,32,45,46,47,48,49] to create differential wettability zones. In this kind of separators, one of the two phases will preferentially wet the porous capillaries or membrane pores while the other phase will be retained. The membrane can be in form of a flat sheet [45] or a tabular membrane [50]. However, in order to ensure complete phase separation, pressure difference across two sides of the capillary or membranes must be controlled within defined limits. Theoretically, the upper limit of the pressure difference is provided by the capillary force, as calculated by Young-Laplace equation, whereas the lower limit is the minimum pressure required to drive the wetting phase through the capillary or membrane pores [27,45,51]. Lu et al. has proved theoretically and experimentally that the pore size distribution also affects these limits [52]. Establishing the pressure difference needed for successful separation can be done either manually or autonomously. The autonomous approach has been a topic of interest because it simplifies its integration with other unit operations, such as flow reactors, without the requirement for continuous adjustments by operators. Some solutions allow such autonomous balance by electronically controlling the transmembrane pressure and regulating the outlets back pressure dynamically to rebalance any variations [53]. Adamo et al developed a self-tuning element in which an elastic polymer film was installed between the two sides, thus dynamically controlled the pressure difference without the need of electronics (Figure 2B) [27,54]. Now commercially available under a trade name of Zaiput (Figure 2C), such separator can easily be implemented as in-line unit [55,56], or multiple units of the separator can be arranged for multistage extraction [57], as shown in Figure 2D. Another approach to autonomously control the pressure drop was recently developed by Bannock who detected light transmissions at the two outlets of the separator and adjusted a needle-valve located downstream of the porous capillary [58]. Other than capillaries and membranes, other designs of separators that utilized surface phenomena can be found in the literature. For instance, Liu et al. constructed a simple phase separator with a hydrophobic T junction and a hydrophilic needle [59], offering an easy-to-assemble setup for laboratory testing.

## 4. Use of LLE for Radioisotopes Production

The Nuclear Medicine community is currently expanding its applications from diagnostic imaging towards therapeutic treatments of several disease (e.g., cancer, metastases, arthritis), thanks to the wide spectra of available radiopharmaceuticals coming from the combinations of targeting bioactive molecules and radionuclides with variable emission properties. The rapid increase in routine applications of diagnostic, therapeutic and theranostics radioisotopes necessitate reliable availability of these medical radioisotopes at reasonable costs.

Research on medical-radionuclide production is a growing interdisciplinary field with final impacts on medicine and the routine use of related radiopharmaceuticals. Currently, the technology advancement in the production of radioisotopes from cyclotron allows for the availability of previously exotic species suitable for diagnostic, therapy and theranostics purposes, thus providing Nuclear Medicine practices the aperture of research and operational fields needed to contribute to more personalized therapies.

**Figure 3:**
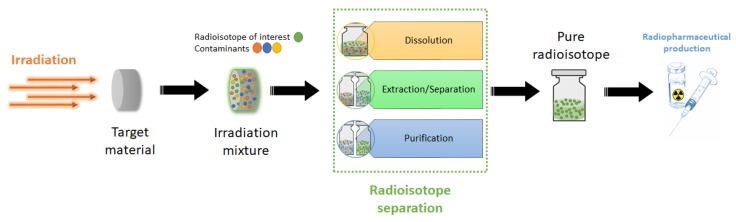
Scheme for the overall radiopharmaceuticals production process. The target material is irradiated to produce the desired radioisotope and, after irradiation, undergoes a radiochemical separation process to isolate the desired pure radioisotope. Such radioactive product is then used to manufacture the final radiopharmaceutical.

Medical isotopes are commonly produced with nuclear reactors or accelerators using processes of fission, neutron capture or bombardment of accelerated particles on a suitable target material. These nuclear reactions can be optimized to achieve selective mass transmutations, resulting in material mixtures containing the desired generated radioisotope. The entirety of chemical steps applied to such produced radioactive mixture to yield a high-purity radioactive species, is referred to as radiochemical separation. This separation represents a fundamental step in the medical production of radioisotopes, as it enables the isolation of the desired radioactive species from different elements, fission products, targets and by-products, in a form suitable for its intended application, as depicted in Figure 3. In this field, a working compromise is always sought among the standard chemical separation methods; however, they are not easily applicable since the conditions are mostly of non-equilibrium, and safe procedures for the treatment of radioactive materials impose certain safety boundaries on separation methods [60]. Indeed, the reactor- or accelerator-produced radionuclide processing has to take place in a shielded and controlled environment, such as a radiochemistry hood or hot cell inside laboratories that are authorized to perform the detention and handling of radioactive tracers and waste [61]. Moreover, if the product of interest is intended for human use, Pharmacopoeia and similar regulatory bodies impose specific product quality requirements for injection, such as sterility, apirogenicity, specific activity, as well as chemical, radiochemical and radionuclidic purity. In addition, final users, as local pharmaceutical manufacturers, will have to comply with additional regulations on the successive radiolabelling process (e.g., in “cold-kit” labelling procedures). Stringent requirements are also typically imposed on the overall procedure time, as this parameter will have to conveniently align with the half-life of the product radionuclide.

In this regard, the employment of automated systems allows for maximizing the reproducibility and final recovery yield, while minimizing operation time, activity losses, human errors and radiation exposure. The setup of the automated process is typically preferred, for both the radiochemical separation and the radiopharmaceutical synthesis, when going from experimental testing levels (i.e., <0.5 GBq) to clinical production levels of activity. Commercial modules are routinely involved in nuclear medicine radiopharmacies for radiopharmaceutical synthesis. These options can be further customized from off-the-shelf options by exploiting modular designs, variable consumable sets and open software systems. This option is fundamental when new processes are designed, or even when known processes must be adapted to the unique needs of the radiochemistry site [62].

A reliable, efficient and safe radiochemical separation process should satisfy many aspects. First, the presence of undesired radioactive emissions (e.g., due to unstable radionuclides) has to be minimized in order to achieve a specific emissive profile in the final product. In addition, the separation from stable isotopes is critical to the success of subsequent radiolabelling. For the same reason, separation processes must avoid the introduction of stable contaminants. The timing needed for efficient separation must be appropriately scaled with the decay rate of the radioisotope of interest, and in general should be as short as possible. Radiological exposure to operators should be minimised, which is typically achieved via automation of processes. The yield of the desired product must be maximized in order to ensure success of subsequent labelling utilizations. Finally, when the radiochemical separation process is tightly linked to the radiopharmaceutical manufacturing, the final product must also satisfy quality requirements for injectability.

Radiochemical separation can be performed by implementing conventional separation methods as standalone unit or as an integrated system; such methods include solid phase extraction (SPE), resin chromatography, liquid-liquid extraction (LLE), precipitation, distillation and sublimation, all of which need to be applied as directly as possible to the irradiation mixture. For this reason, the separation procedure is often individually customized for each application since it is highly sensitive to the physical and chemical properties of the target material (e.g., liquid, solid or gas) and the physical and chemical properties of the desired radionuclide.

Although all the separation methods mentioned above are important in the radiochemical separation field, LLE is particularly well suited to the purification of many radioisotopes. This technique is based upon the selective partitioning of solutes between two immiscible solvent phases, usually an aqueous solution (i.e., acids, bases or salts) and an organic solvent (e.g., ethers, amines and ketones), while controlling pH, phase volumes, mixing time and aqueous phase composition. It provides a high selectivity even when dealing with very low concentrations, as partition conditions can be easily varied to obtain radioisotope yields relatively free from chemical and radionuclidic impurities. LLE can be performed rapidly (a particularly vital characteristic when isolating isotopes with shorter half-lives), and is relatively simple to automate [61,63]. Typically, LLE is followed by evaporation of the extracting solvent, back extraction in aqueous phase or SPE/chromatography to purify the radionuclide from the organic phase and dissolved into a solution phase appropriate for successive radiolabelling or injection.

Among the variety of separation techniques employed for the radiochemical isolation and purification of nuclides, separations by LLE and resin chromatography are the most widely used. Both techniques are simple, convenient, fast and clean. However, since most chromatography equipment are readily automated, LLE processes have been used only in research tests without breaking the market of automated synthesis modules. However, LLE has historically been used at the inception of medical radioisotopes production, methylethylketone (MEK) based solvent extraction technique was applied in the first examples of ^99m^Tc productions, as reported by Gerlit in 1955 [64] and later on employed in several generator apparatuses that gained widespread use [65,66]. A review published by Navratil investigated the use of LLE methods to extract radionuclides of interest in nuclear technology [67]. In the review, the importance of phosphate-based ligands was highlighted due to the flexibility of use achievable by molecular modification. Relevant to radiopharmaceuticals, pioneering works of Lahiri et al reported [68] the separation of Tc and Ru isotopes from α-particle activated Mo (various isotopes) using organic solutions of di-(2-ethylhexyl)phosphoric acid (HDEHP) and tributyl phosphate (TBP) as extractants.

In this section we will provide recent examples of the use of LLE separation of clinically relevant radioisotopes (Table 1).

The separation of ^67^Cu, an emergent theranostic tracer, is currently unavailable at routine level. Its cyclotron production from zinc target is now under investigation [69], while LLE approaches have been reviewed by Smith et al. [70], reporting examples applicable to cyclotron or accelerator produced ^67^Cu. Therein, the target material is represented by a Zn salt that, after irradiation, is dissolved in 0.5 M HCl. The most widely utilized liquid-liquid extraction procedure involves a first step of Cu separation from the target solution using a 0.01% solution of dithizone in CCl_4_ (this is repeated 4 times) followed by a back-extraction of Cu from the organic fraction using a 7.2 M HCl and H_2_O_2_, and removal of Ga contaminants via isopropylether LLE. Additional metal impurities, such as Ni, Mn, Cr, and Co, can be removed by passing the product solution through an anion exchange column [71,72,73,74]. It is worth noting that alternative organic extractants have been reported by other authors, such as 8-mercaptoquinoline in toluene [75], thenoyltrifluoroacetone in benzene [76] and a 1% solution of resacetophenone oxime (RAPOX) in cyclohexane [77].

Pietrelli et al [78] reported in 1992 a preliminary study on using LLE to separate ^47^Sc, another emergent theranostic tracer, from hydrochloric solutions of Ti target material. The main extraction step involves removal of Ti(IV) by complexation with cupferron (ammonium salt of N-nitrosophenylhydroxylamine) in pure chloroform (this is repeated 5 times). Alternatively, Sc solvent extraction could be realized by using tri-n-butyl phosphate (TBP) ligand, according to a preferential solvation reaction with Sc. However, given the large difference in concentrations between Ti and Sc, a successful use of such approach would require multiple extractions. A way to improve this method is to support the ligand by impregnating TBP into a SiO_2_ support, and use column chromatography [79]. Similar phosphate compounds are available as supported reagents (UTEVA resin) and have been successfully used for the separation of ^44^Sc from cyclotron irradiated Ca targets [80]. However, currently no LLE extraction methods have been reported for the extraction of ^44^Sc, even though very efficient separations are obtained between Sc and Ca in non-radioactive industry, also using phosphate-based ligands [81].

The separation of ^89^Zr, employed in imaging studies with slowly-accumulating bioactive molecules such as immuno-PET with monoclonal antibodies [89], from yttrium targets using LLE has been reported by Kandil et al [83]. After cyclotron bombardment, the yttrium oxide target material was dissolved into 9 M HCl at 100 °C in 30 min. Extraction of Zr was performed using an equal volume of 3% (*w*/*v*) TPPO (triphenylphosphine oxide) in chloroform solution; the organic phase was then back-extracted in aqueous phase by adding 0.5% oxalic acid producing zirconium oxalate [83]. This solution was evaporated and reconstituted with 0.1 M citrate buffer at pH 4. It is worth noting that in this study, di-(2-ethylhexyl) phosphoric acid (HDEHP) was also tested as organic extractant, but the inability to back-extract Zr into the aqueous phase as pure inorganic salt rendered this approach unfeasible for radiolabelling purposes.

The LLE separation of ^52^Mn, a radionuclide suitable for multimodal PET-MRI imaging having both paramagnetic and proper nuclear properties, from chromium target has recently been reported. The target material consisted of Cr dissolved in HCl 9 M which underwent LLE separation employing 0.8 M trioctylamine (TOA) diluted in cyclohexane as an organic extractant for Mn. Back-extraction was performed using 5 mM NH_4_OH and three cycles of this purification were performed to ensure optimal purity [84,85]. The ammonium solution was further passed through a C-18 resin (to reduce the contamination from organics), dried and reconstituted with PBS. The resulting solution could be used for successive PET in vivo studies.

Another interesting concept in LLE is the use of ionic liquids (IL), which are species whose solubility can be tuned to be insoluble with aqueous or organic phases. The used of IL-mediated LLE has been tested in the separation of ^225^Ac/^227^Th nitric acid sample mixture. A series of IL was investigated [86], using *N*,*N*,*N*′,*N*′-tetraoctyldiglycolamide (TODGA) or di(2-ethylhexyl) phosphoric acid (HDEHP) as dissolved extractants. All these tests were conducted using the same LLE process, but varying the HNO_3_ and extractant concentration and the IL identity. TODGA proved to be very efficient in extracting both metals out of the aqueous phase, but did not provide acceptable separation between the two metals in any of the conditions tested. On the other hand, HDEHP resulted to be a much better extractant of Th compared to Ac, especially at higher acidities, providing a separation factor >10^7^ at 0.01 M HNO_3_ using the appropriate combination of IL and HDEHP concentration. This study could open the way to new methods to purify ^225^Ac, a radionuclide gaining increased visibility due to the interest in α-therapy regimens.

In the past decade, there are two examples of LLE-based automatic modules for the extraction, separation and purification of ^99m^Tc, still the most widely used radionuclide worldwide in Single Photon Emission Computed Tomography (SPECT). 

**Figure 4:**
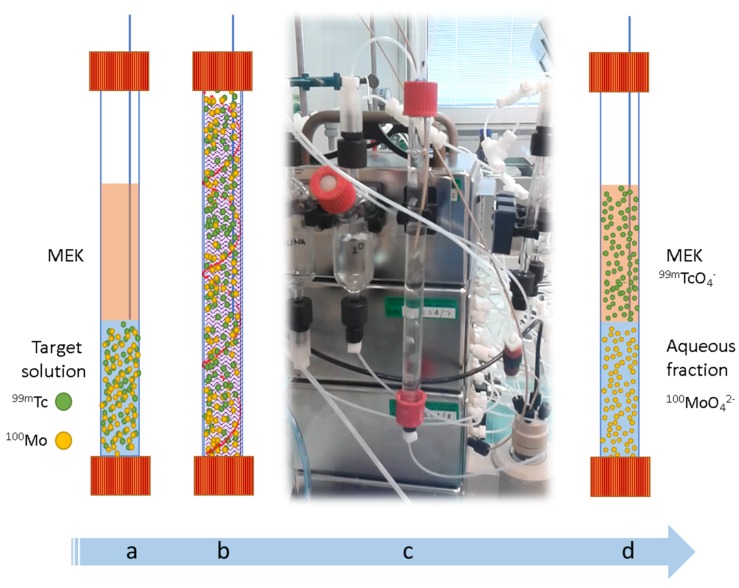
Schematic description of the steps of the automated on-column solvent extraction with MEK of ^99m^Tc from a ^100^Mo irradiated target solution. (**a**) addition of the extractant MEK to the aqueous target solution containing molybdate, pertechnetate anions and contaminants; (**b**) mixing of the two immiscible phases through helium bubbling and extraction of the pertechnetate by the solvent MEK from the aqueous phase; (**c**) photograph of the bubbling step in the automatic module developed by Martini et al; (**d**) phases separation.

Both modules utilize methyl ethyl ketone (MEK) as extractant for Tc, but while the first module was custom made for low-medium specific activity ^99^Mo/^99m^Tc decay-extraction process, the second system was designed for processing a cyclotron-irradiated ^100^Mo target, and obtained by assembling commercially available modular units [87,88,90,91,92]. In this example, the automation of the LLE started with a ^100^Mo irradiated target dissolved in H_2_O_2_ and 6 M NaOH, and this aqueous solution was mixed with neat MEK into a contacting column (Figure 4a). Increased extraction was achieved by bubbling inert gas or air in the separation column for few minutes (i.e., 7 min) by maximizing the interfacial surface area between the two immiscible layers (Figure 4b,c). Once the desired extraction equilibrium was reached (Figure 4d), the mixture was allowed to settle (i.e., 5 min) and a fixed capillary placed above the interface used to recover the Tc-rich organic phase on top.

Additional MEK washes were needed to increase recovery and the MEK solution was passed through a silica column in order to enable the solvent switch to aqueous phase, utilizable in successive labelling processes. Detailed qualitatively evaluation demonstrated that the final product basically meets the quality requirements recently issued by the European Pharmacopeia monograph on cyclotron produced ^99m^Tc [91,93,94].

The same approach was recently applied by Capogni et al [95] using a semiautomatic module for the extraction of low specific activity ^99m^Tc-pertechnetate from neutron activated ^99^Mo.

The tendency towards miniaturization nowadays is driving the development of micro-scale reactors (lab-on-chip) for applications in radiochemical separation to improve performance and minimize chemical and radiological hazards, the two major hazards in the radiopharmaceutical field. Among current radiochemical separation systems, LLE could be the most suitable for the miniaturized scale. However, knowledge on the microscale interfacial dynamics and kinetics as well as optimization of the extraction microenvironment are necessary for future evolution in the radiochemical separation field [33]. 

Few examples of miniaturization of LLE have been reported, as in the case of a study [33] on the use of different microfluidic geometries for achieving radioisotope separations towards analytical applications or the design of a novel slit-length design SIFEL-based platform used for co-current extraction of 0.1 M HCl ^64^Cu solution with a toluene solution of 2-hydroxy-4-*n*-ctyloxybenzophenone oxime (HOBO) [96].

**Figure 5:**
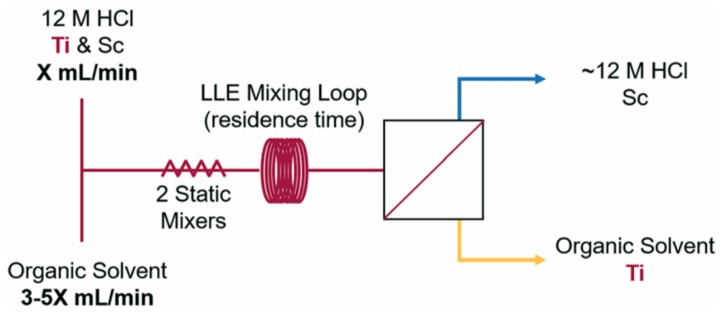
Instrumental set-up used in the flow LLE and separation of Ti from Ti/Sc mixture.

Recently, an innovative platform for the LLE separation of ^45^Ti from a cyclotron irradiated ^nat^Sc was demonstrated. It employed a continuous flow extraction and membrane-based phase separation as depicted in Figure 5 [41]. In this work, the Sc foil target material was dissolved in 12 M HCl and extracted with the solution of organic extractant, either in flow or batch environment. Preliminary non-radioactive experiments identified a 9:1 (*v*/*v*) guaiacol/anisole mixture as the best extractant for ^45^Ti, and this system was used to compare batch and flow extraction. Additionally, to modify the nature of contacted solutions, few parameters were optimised in the batch protocol (e.g., shaking time), while several modified conditions were achievable in flow extraction by varying mixing time, but also tube diameter, flow rate, flow ratios, presence of passive mixers.

In addition, the separation was conducted in flow, using a Zaiput membrane separator and an appropriate choice of membrane pore size was required. The optimal conditions resulted in >80% extraction of ^45^Ti from irradiated target material in a single flow LLE process. As a proof of principle, such organic solution was collected and directly reacted with an equimolar amount of salan and 2,6-pyridinedicarboxylic acid (dipic) in pyridine at 60 °C, to obtain nearly quantitative conversion to [^45^Ti](salan)Ti(dipic), a Ti-antineoplastic previously reported for pre-clinical PET imaging.

## 5. Conclusions

The increasing demand of more personalized treatments is catalysing a considerable number of developments to provide clinical benefits. Originally limited to nuclear fuel cycle research, the separation and purification of radioactive isotopes is gaining a growing importance in Nuclear Medicine, due to the nearly endless flexibility of half-lives, chemistries and radioactive decay modes that could fit different treatment scenarios. Despite their use in historical radioisotopes separations, LLE methodologies have been largely substituted by resin-based approaches, which could be more easily automated. Large scale LLE approaches are currently used in hydrometallurgy field, where larger quantities of materials are processed compared to the medical radioisotopes case (ML vs mL); therefore, there is a vast body of knowledge available from this field that has not yet been fully exploited in the radiometals field.

On the other hand, recent developments in flow chemistry has demonstrated how extraction processes can be achieved with much better control and efficiency by exploiting the unique features of flow regimes, that can be scaled up or down without major re-optimization issues. In addition, flow phase separation is now a reality implemented in several simple prototypes and commercially available system, allowing a straightforward phase separation of continuously flowing multi-phasic mixtures.

If taken together, this information points towards the high likelihood of future LLE processes for purifying medically relevant isotopes that utilizes extensively flow chemistry approaches. Indeed, this trend has been demonstrated by the first reported publication for the purification of ^45^Ti from ^nat^Sc irradiated target, performed using flow regime for both extraction and phase separation. We think that this example will allow radiochemists to surpass the view of radiometals LLE as only a shake-flask system that is difficult to control and automate, and move towards more up-to-date flow systems.

The trend of innovations, overviewed in this review, predicts that LLE approaches will coexist with currently used resin-based methods. The ease of testing of LLE systems, especially when implemented in flow, will be very important when studying new separations, while the availability of stable resin-based processes will be relevant for implementing known separations. It is obvious that both these approaches have different advantages and drawbacks, and the choice will be dependent on experience, equipment and financial feasibility. However, the possibility of easy scaling and the inherently cheaper nature of LLE will likely increase its interest, especially due to the latest automation possibilities offered by flow systems.

## Figures and Tables

**Table 1:** Synoptic table of example medical interest radioisotopes purified via LLE methods.

Radiometal Extracted	Originating Mixture	Extraction Solvent	% of Extraction (LLE Repeats, Number)	Notes	Reference
^67^Cu	Zn salt dissolved in 0.5 M HCl	0.01% dithizone in CCl_4_	(4)	back-extraction in 7.2 M HCl/H_2_O_2_ + purification from Ga and metal contaminants via isopropylether LLE + anion exchange column	[71,72,73,82]
		8-mercaptoquinoline in toluene	not reported		[75]
		thenoyl-trifluoroacetone in benzene	~90%	back-extraction	[76]
		1% solution of resacetophenone oxime (RAPOX) in cyclohexane	not reported		[77]
^47^Sc	hydrochloric solutions of Ti	cupferron in pure chloroform	(5)	Extraction by Ti(IV) complexation	[78]
		tri-*n*-butyl phosphate (TBP)	(multiple)	Alternative: impregnating TBP into a SiO_2_ support, and use column chromatography	[79]
^89^Zr	Y_2_O_3_ in 9 M HCl	3% (*w*/*v*) TPPO in chloroform	not reported	zirconium oxalate back-extraction in 0.5% oxalic acid	[83]
^52^Mn	Cr	0.8 M TOA in cyclohexane	(3)	Back-extraction in 5 mM NH_4_OH	[84,85]
^225^Ac/^227^Th	nitric acid sample mixture	Ionic Liquid/TODGA	not reported	unacceptable separation of Th and Ac	[86]
		Ionic Liquid/HDEHP	not reported	Th separation factor >10^7^ at 0.01 M HNO_3_	[86]
^99m^Tc	[^100^Mo] Na_2_MoO_4_ in 6 N NaOH	MEK	93 ± 3% (2)	Further Purification by chromatographic columns	[87]
	[^99^Mo] Na_2_MoO_4_ in 5 N NaOH	MEK	78–90% (multiple, generator-like decay-extractions)	Further Purification by chromatographic columns	[88]
^45^Ti	Sc in 12 M HCl	9/1 (*v*/*v*) guaiacol/anisole mixture	>80% (1)	Continuous flow extraction and membrane phase separation	[41]
